# An Atypical Skin Presentation of Adult‐Onset Still's Disease With Histological Description: A Case Report

**DOI:** 10.1111/cup.70155

**Published:** 2026-06-18

**Authors:** M. Nordmann, T. Mazza, S. Menzinger, G. Kaya, S. Bogiatzi, S. Vandenberghe‐Dürr, M. Blanchard, J. Stirnemann

**Affiliations:** ^1^ Division of Internal Medicine Geneva University Hospitals Geneva Switzerland; ^2^ Division of Clinical Pathology Geneva University Hospitals Geneva Switzerland; ^3^ Division of Dermatology Geneva University Hospitals Geneva Switzerland; ^4^ Division of Allergology and Immunology Geneva University Hospitals Geneva Switzerland

**Keywords:** skin, still's disease

## Abstract

Adult‐onset Still's disease (AOSD) is an auto inflammatory disorder with a variable clinical presentation, and without a pathognomonic diagnostic test, characterized by high spiking fever, arthralgia/arthritis, a suggestive skin rash, elevated white blood cell count 10 G/L (> 80% neutrophils), elevated ferritin, with glycosylated ferritin < 20% and high IL‐18. Cutaneous manifestations are very frequent and the commonest description is an evanescent salmon‐pink rash, although many atypical skin presentations have been described in literature. We present the case of an atypical cutaneous manifestation of clinically typical AOSD in a 30‐year‐old man presenting with a slightly pruritic maculopapular and urticarial erythematous rash on the face and limbs, with purpuric features on the lower limbs, which gradually spread to the whole body, associated with overlying nonfollicular pustules. The patient also presented with high spiking fever, odynophagia, diffuse arthralgia, and generalized asthenia. The histological analysis of a skin biopsy showed a few subcorneal collections of neutrophils, a slight epidermal spongiosis, many suprabasal necrotic keratinocytes, and a moderate mixed inflammatory infiltrate in the superficial dermis. Following various investigations ruling out any other diagnosis, we have diagnosed Still's disease, for which the patient has been prescribed corticosteroids, with good initial results.

## 1 Introduction

Adult‐onset Still's disease (AOSD) is an autoinflammatory disorder with variable clinical presentation but no pathognomonic diagnostic test. The Yamaguchi criteria include high fever spikes (≥ 39°C), arthralgia/arthritis for 2 weeks, typical rash and white blood cell count 10 G/L (> 80% neutrophils) [1]. Minor criteria include sore throat, lymphadenopathy, hepatomegaly or splenomegaly, abnormal liver function tests, and the absence of rheumatoid factors and antinuclear antibodies (ANA) [2]. Exclusion criteria are necessary and include infections, malignancy, and other rheumatic diseases. Main biological findings comprise elevated ferritin [3], with glycosylated ferritin< 20% [3, 4], negative rheumatoid factor and ANA. Serum IL‐18 (cutoff > 18 ng/mL) helps differentiate AOSD from sepsis and secondary hemophagocytic lymphohistiocytosis (HLH) [5]. AOSD is usually a benign, nonfatal disease, although sometimes more serious complications can occur like macrophage activation syndrome (MAS), or disseminated intravascular coagulation [3]. Corticosteroids are the first‐line treatment, which can be combined with other immunosuppressive therapies. Cutaneous manifestations, including the typical salmon‐pink and evanescent rash, are important criteria in AOSD [6]. Cutaneous manifestations are non‐specific and can be misdiagnosed as urticaria, viral exanthema, or cutaneous drug reaction, due to AOSD being a diagnosis of exclusion [6]. We present a case of confirmed AOSD with atypical cutaneous presentation.

## 2 Case Report

A 30‐year‐old man initially presented with odynophagia, and a slightly pruritic skin rash appearing on his face and feet on day two, spreading gradually to the rest of his body the following day. The initial presentation was a maculopapular and urticarial erythematous rash of the face, trunk, and limbs (Figure 1), slightly pruritic, with purpuric features of the lower limbs, gradually spreading to the whole body and associated with overlying nonfollicular pustules (Figure 2). The lower limb lesions were more erythematous and infiltrated, not migrating, with fluctuating infiltration and redness. Mucosal involvement was absent. He had not taken over‐the‐counter medications or supplements before this first eruption. A hyperthermia was noted from the fourth day with a daily peak of 40°C, associated with diffuse arthralgia and myalgia without clinical arthritis lasting several weeks, adenopathy, and asthenia. Laboratory investigations showed a high C‐reactive protein (312 mg/L; normal value [nv] < 5 mg/L), leukocytosis (36 G/L; nv 4–11 G/L) with non‐segmented forms (neutrophils 32 G/L; nv 1.5–7.5 G/L), altered liver tests (mild cytolysis and cholestasis) with normal bilirubin levels, an elevated LDH of 506 U/L (nv 87–210 U/L), slightly elevated proBNP (395 ng/L; nv < 300 ng/L), an initial ferritin of 3670 μg/L (nv 11–342 μg/L), subsequently rising to > 59 000 μg/L, non‐calculable glycosylated ferritin with a percentage of glycosylation < 19% (nv 50%–80%), and high triglycerides (3.02 mmol/L; nv < 1.7 mmol/L). Renal function, hemoglobin, creatine kinase, coagulation, and procalcitonin levels were within normal limits. Screening for antiphospholipid antibody syndrome and autoimmune diseases proved negative: Immunoglobulins, cryoglobulin, C3/C4 complement, ANA, anti‐nucleosome antibodies, anti‐nuclear cytoplasmic antibodies (ANCA), rheumatoid factor, anti‐streptolysin O antibodies, anticoagulant lupus, anticardiolipin antibodies, anti béta2‐glycoprotein. Extensive microbiological samples were negative. Immunofixation monoclonal immunoglobulin was absent. Free IL‐18 was markedly elevated (37.5 ng/mL), consistent with the diagnosis of Still's disease.

**FIGURE 1:**
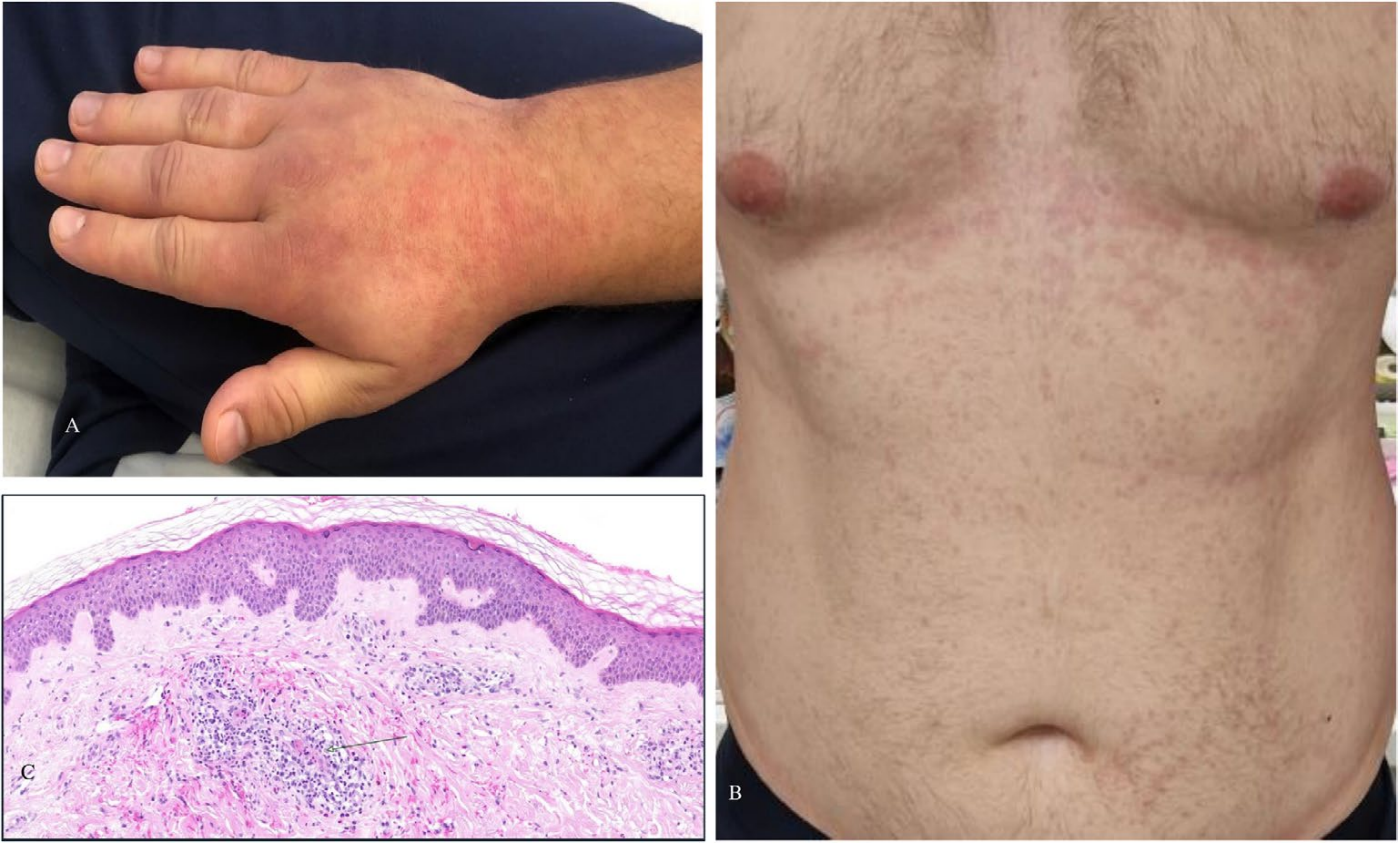
A, B and C. Eruption 1. (A) (Hand) and (B) (Trunk): Initial maculopapular and urticarial erythematous rash. (C) Biopsy 1. Hematoxylin and eosin stain, original magnification ×150. A slight superficial perivascular inflammatory infiltrate composed of lymphocytes, with very few neutrophils and eosinophils, without signs of leucocytoclasia, and associated with red blood cell extravasation and vascular wall fibrinoid necrosis (arrow).

**FIGURE 2:**
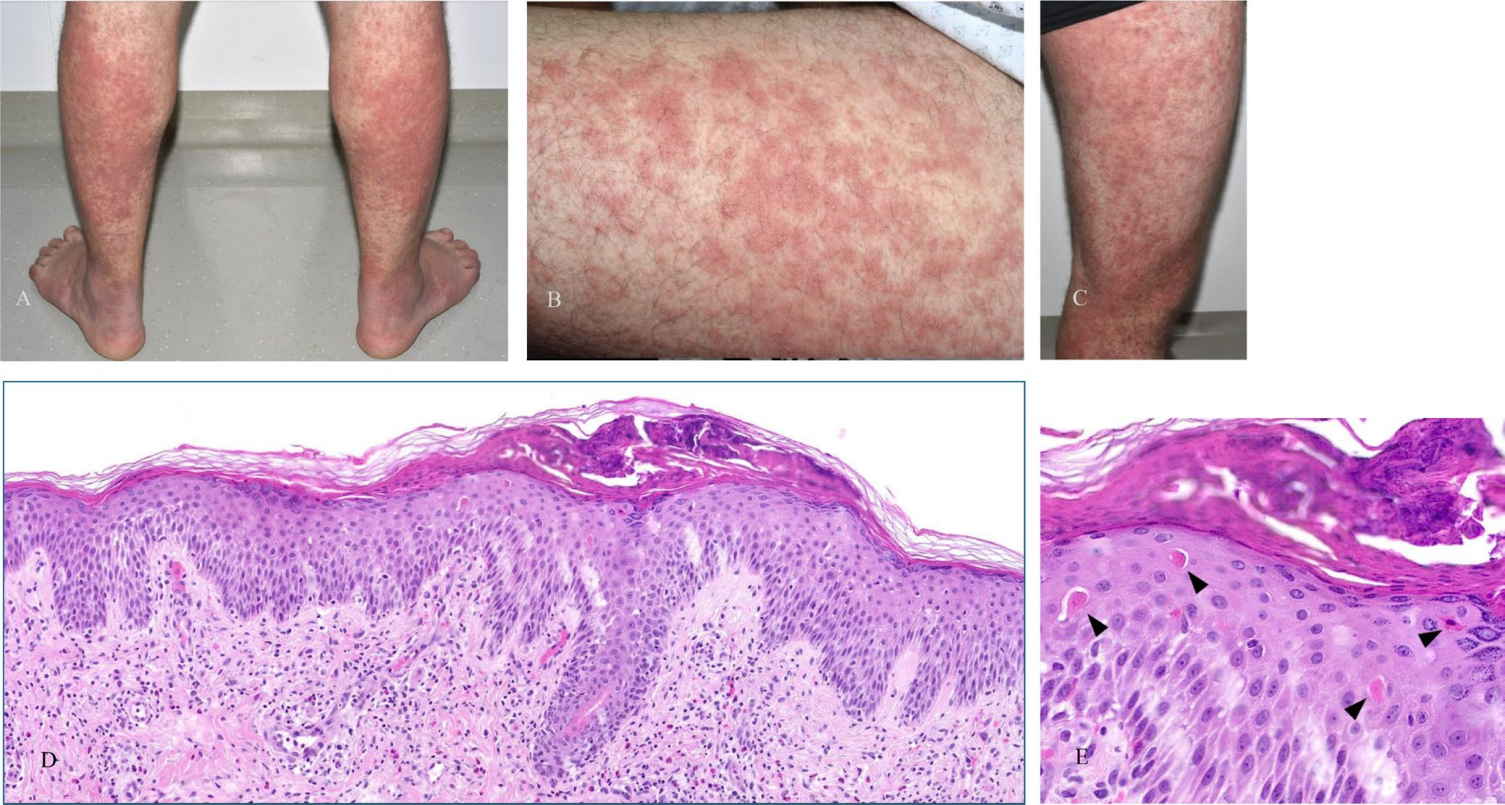
A, B, C, D, and E. Eruption 2: Posterior legs and feet: Erythematous maculopapular rash, purplish in places, confluent in plaques with ill‐defined crumbled edges. (A) Purpuric appearance on the feet. (B) External thigh: Erythematous maculopapular rash confluent in plaques with poorly defined crumbled edges. (C) Thigh: Erythematous maculopapular rash confluent into a plaque and topped by small pustules. (D) and (E) Biopsy 2. Hematoxylin and eosin stain, original magnification ×150. Parakeratosis with intracorneal collection of neutrophils, many suprabasal necrotic keratinocytes, a slight spongiosis, and a mixed inflammatory infiltrate in the superficial dermis with few neutrophils and numerous eosinophils.

Histology of the first skin biopsy from the left lower leg (Figure 1) showed lymphocytic thrombosing vasculitis. The differential diagnoses included viral exanthema, rickettsial infection (Mediterranean spotted fever), drug reaction, or possibly a serum sickness disease. A second biopsy (left thigh, Figure 2) showed subcorneal neutrophil collections, slight epidermal spongiosis, suprabasal necrotic keratinocytes, and moderate mixed inflammatory infiltrate (lymphocytes, histiocytes, eosinophils, and neutrophils) in the superficial dermis. Though non‐specific, this pattern can be consistent with AOSD. After excluding infectious and autoimmune disease, and solid or hematologic malignancies, AOSD was diagnosed per Yamaguchi criteria: three to four major criteria (arthralgia, fever, and neutrophilia> 10 G/L) and four of the five minor criteria (sore throat, lymphadenopathy, abnormal liver function tests, negative RF and ANA).

After an oral prednisone therapy of 80 mg, symptoms resolved with an improvement in blood values after 1 week. The vasculitis‐like lesions of the feet improved, but the lower limbs rash evolved into infiltrated erythematous plaques with many overlying small non‐follicular pustules. After hospitalization, steroids were gradually reduced over several months and cutaneous lesions disappeared. The patient remains stable since discontinuation of corticosteroid therapy 6 months ago and continues treatment of 1 mg of colchicine daily.

## 3 Discussion

Still's rash is typically a salmon‐colored, nonpruritic, evanescent macular/maculopapular eruption, predominantly of the trunk and extremities, occasionally involving palms, soles, and face, coinciding with fever. The Koebner phenomenon may be present [1, 7]. Histopathology is non‐specific and usually shows a perivascular mixed infiltrate in the upper dermis, with a variable number of neutrophils, rarely with leukocytoclasis, and without vascular wall fibrinoid necrosis. The epidermis is usually unaltered, and eosinophils are usually absent [8, 9, 10]. Although this presentation is non‐specific [9, 10, 11, 12], it is useful to rule out other diseases.

Other documented cutaneous manifestations include persistent papules/plaque eruptions (PPE), often pruritic, erythematous to violaceous, on the trunk and extensor extremities, sometimes associated with scales or crusts [6, 8]. The six most frequent of the different observed morphologic patterns are PPE with linear configuration [1, 10], urticarial papules, erythematous to violaceous maculopapules or patches in clear photo distribution (dermatomyositis‐like), prurigo pigmentosa‐like eruption, lichenoid papules or plaques, and lichen amyloidosis‐like [1, 11, 13]. The PPE with linear configurations is commonly found and is indicative of the Koebner phenomenon [11], resembling a flagellate erythema [14, 15]. Histopathology shows multiple individual or aggregates of necrotic keratinocytes in the upper epidermis with focal hyperkeratosis, sometimes with increased dermal mucin, associated with a sparse infiltrate in the upper dermis composed of neutrophils and lymphocytes, but without signs of vasculitis [8, 11, 16, 17]. The persistent erythematous to violaceous maculopapules or patches are pruritic, dermatomyositis‐like, with a clear photodistribution, often associated with swelling or erythema of the upper eyelids, and erythematous maculopapules on the fingers like Gottron papules [1, 11]. The prurigo pigmentosa‐like eruption is severe pruritic, red papules that may tend towards brownish lichenoid papules, with a reticulate pattern on the chest and upper back [1, 12]. These five atypical persistent pruritic eruptions are characterized by necrotic keratinocytes in the upper epidermis (and horny layer in developed lesions), with a mixed (lymphocytic and neutrophilic) superficial perivascular or interstitial infiltrate. Spongiosis, basal vacuolar degeneration, and epidermal exocytosis of inflammatory cells are rare [8, 11, 16, 17]. However, the typical AOSD rash shows normal epidermis and a sparse perivascular infiltrate of lymphocytes and neutrophils in the upper dermis, which is less abundant than in the PPE. Rare eosinophils found in atypical AOSD are absent in typical AOSD [11].

Neutrophilic urticarial dermatosis (NUD) is an autoinflammatory condition characterized by an urticarial rash vanishing in 24–48 h, almost always accompanied with systemic symptoms as arthralgia or fever. Histologically, there is a dense neutrophilic interstitial infiltrate in the dermis and a leukocytoclasia in association with dyskeratotic/necrotic keratinocytes, without damage to the vessel walls. NUD is part of neutrophilic disease, not of the urticaria group, and this condition is strongly associated with systemic diseases, like Still's disease [18, 19, 20, 21].

AOSD predisposes to HLH, a pathologic immune activation, potentially leading to organ failure. HLH was considered given high‐grade fever, hypertriglyceridemia, and markedly elevated ferritin, but criteria were not met due to the absence of cytopenia, splenomegaly, or hypofibrinogenemia. The American College of Rheumatology recommends IL‐18 as a specialized biomarker to help differentiate Still's disease (with or without MAS) from other forms of HLH/MAS, given its relative specificity and diagnostic utility [22]. Soluble CD25 was not measured, neither was a bone marrow aspiration performed due to the rapid favorable response to systemic corticosteroid alone [23].

Other pathologies with similar skin lesions or histopathology [11], include Henoch Schönlein disease [24], acute generalized exanthematous pustulosis [25], dermatomyositis [26], lichen planus [27], drug eruption and viral exanthem [28], familial Mediterranean fever [29], Sweet syndrome [30] and others. Although analyses were negative, the initial eruption being caused by a virus, which triggered the AOSD is another hypothesis.

In conclusion, AOSD can be diagnosed with near‐certainty when clinical, biological, and histopathological findings converge. Specifically, the presence of many scattered dyskeratotic/necrotic keratinocytes in the upper portion of the epidermis associated with a neutrophilic infiltrate is a finding not seen in many entities. In our patient, IL‐18 provided strong confirmation of the diagnosis of AOSD, possibly associated with HLH, which was controlled with corticosteroid therapy [22, 31].

Diagnosing the disease remains a matter of exclusion for which it is essential to pool clinical, histopathological, and laboratory data, and this is where suggestive skin lesions may be absent.

It is certain that many diagnoses have identical clinical and/or histopathological characteristics, making it necessary to link both the clinical manifestations of the skin lesions and the histopathological results available in order to make the final diagnosis.

## Author Contributions

M.N. and J.S. prepared the first draft of the manuscript; M.B. obtained cutaneous images; S.M., G.K., and S.B. obtained scanned histological images. All authors (MN, SM, GK, SB, TM, SVD, JS) were involved clinically in the case and provided editorial support throughout the preparation of the manuscript.

## Ethics Statement

Informed, written consent was obtained from the patient for publication of this manuscript, and all efforts have been made to anonymize any personal identifying information.

## Conflicts of Interest

The authors declare no conflicts of interest.

## Data Availability

Data sharing not applicable to this article as no datasets were generated or analysed during the current study.
